# Tripsazea, a Novel Trihybrid of *Zea mays*, *Tripsacum dactyloides*, and *Zea* *perennis*

**DOI:** 10.1534/g3.119.400942

**Published:** 2019-12-02

**Authors:** Xu Yan, Mingjun Cheng, Yingzheng Li, Zizhou Wu, Yang Li, Xiaofeng Li, Ruyu He, Chunyan Yang, Yanli Zhao, Huaxiong Li, Xiaodong Wen, Ping Zhang, Ebenezer Sam, Tingzhao Rong, Jianmei He, Qilin Tang

**Affiliations:** * Maize Research Institute, Sichuan Agricultural University, Chengdu 611130, China,; † Animal Husbandry Research Center,; ‡ Sericulture Research Institute, Sichuan Academy of Agricultural Sciences, Nanchong 637000, China,; § Sichuan Grass Industry Technology Research and Promotion Center, Chengdu 610041, China,; ** Guizhou Prataculture Institute, Guiyang 550006, China, and; †† Institue of Forestry and Pomology, Neijiang Academy of Agricultural Sciences, Neijiang 641000, China

**Keywords:** *Zea mays*, *Tripsacum dactyloides*, *Zea perennis*, trihybrid, maize improvement, forage breeding

## Abstract

A trispecific hybrid, MTP (hereafter called tripsazea), was developed from intergeneric crosses involving tetraploid *Zea mays* (2*n* = 4*x* = 40, genome: MMMM), tetraploid *Tripsacum dactyloides* (2*n* = 4*x* = 72, TTTT), and tetraploid *Z*. *perennis* (2*n* = 4*x* = 40, PPPP). On crossing maize-*Tripsacum* (2*n* = 4*x* = 56, MMTT) with *Z*. *perennis*, 37 progenies with varying chromosome numbers (36-74) were obtained, and a special one (*i.e.*, tripsazea) possessing 2*n* = 74 chromosomes was generated. Tripsazea is perennial and expresses phenotypic characteristics affected by its progenitor parent. Flow cytometry analysis of tripsazea and its parents showed that tripsazea underwent DNA sequence elimination during allohexaploidization. Of all the chromosomes in diakinesis I, 18.42% participated in heterogenetic pairing, including 16.43% between the M- and P-genomes, 1.59% between the M- and T-genomes, and 0.39% in T- and P-genome pairing. Tripsazea is male sterile and partly female fertile. In comparison with previously synthesized trihybrids containing maize, *Tripsacum* and teosinte, tripsazea has a higher chromosome number, higher seed setting rate, and vegetative propagation ability of stand and stem. However, few trihybrids possess these valuable traits at the same time. The potential of tripsazea is discussed with respect to the deployment of the genetic bridge for maize improvement and forage breeding.

In the tribe Maydeae (family Gramineae), maize (*Zea mays* ssp. *mays*) (2*n* = 2*x* = 20Zm, genome = MM) from the genus *Zea* is the most prominent and ubiquitous species due to its high grain yield, forage yield and nutritive value for human food and animal feed as well as its use as an industrial material (Shiferaw *et al.* 2011). However, domestication has deleted many characters due to only breeding for yield and quality, leading to a narrow genetic base of modern maize, which results in increased vulnerability to biotic and abiotic stresses. Fortunately, its wild relatives, *viz*., teosinte and *Tripsacum dactyloides* (2*n* = 4*x* = 72Td, TTTT), are crossable with maize and are considered rich sources of untouched genes for these traits. The use of teosinte and *T. dactyloides* in maize improvement has been extensively reviewed by Harlan and De Wet (1977), Warburton *et al.* (2017), and Mammadov *et al.* (2018). In general, once a genetic bridge is established by crossing maize with teosinte or *T*. *dactyloides*, introgression of resistance to biotic and abiotic stresses possessed by wild relatives through backcrossing the genetic bridge with maize is commonly used due to male infertility and female subfertility of the genetic bridge.

Maize can be crossed with all teosinte species, including *Z*. *mays* ssp. *mexicana*, *Z*. *mays* ssp. *parviglumis*, *Z*. *luxurians*, *Z*. *diploperennis* and *Z*. *perennis*, with conventional breeding methods, although the degree of success is species-dependent (Tang *et al.* 2005; Hufford *et al.* 2012; Warburton *et al.* 2017). The only tetraploid teosinte species is *Z*. *perennis* (2*n* = 4*x* = 40Zp, PPPP); the others are diploid species. The teosinte bridge (mazie × teosinte) has been proven to be feasible for widening the genetic diversity and conferring some resistance to adverse environments in the process of maize improvement (reviewed by Mammadov *et al.* 2018). Adding a teosinte (*Z*. *parviglumis*) rare allele (*Upright Plant Architecture2*, which was deleted during maize domestication) to maize could significantly increase maize planting density and yield by reducing the maize leaf angle (Tian *et al.* 2019). This suggests that maize improvement could benefit from the useful traits hidden in its wild relatives. However, it was quite difficult when maize was pollinated by *T*. *dactyloides* and was only effective in some crosses of maize × *T*. *dactyloides* when the embryo rescuing technique was utilized. Several types of *Tripsacum* bridges have been used to broaden the genetic diversity of maize. Mangelsdorf and Reeves (1931) obtained the first *Tripsacum* bridge (2*n* = 2*x* = 10Zm + 18Td, MT) from the intergeneric cross of diploid *Z*. *mays* × diploid *T*. *dactyloides* by hand crossing and embryo rescue. These authors and De Wet *et al.* (1970) developed the second type of *Tripsacum* bridge (2*n* = 3*x* = 10Zm + 36Td, MTT) from a cross of diploid *Z*. *mays* × tetraploid *T*. *dactyloides*. Kindiger *et al.* (1996a) and Molina *et al.* (2006) reported the development of the third type of *Tripsacum* bridge (2*n* = 4*x* = 20Zm + 36Td, MMTT) from a cross between tetraploid *Z*. *mays* and tetraploid *T*. *dactyloides*. Backcrossed derivatives of three types of *Tripsacum* bridges with maize are also *Tripsacum* bridges and the only pathway to improve maize by *Tripsacum*. Eubanks (1995) reported a controversial *Tripsacum* bridge (Bennetzen *et al.* 2001) generated from crossing *T*. *dactyloides* (2*n* = 36 or 2*n* = 72) with *Z*. *diploperennis* (2*n* = 20), displaying 20 somatic chromosomes, regular synapsis in pollen mother cells (PMCs), and high pollen fertility.

To date, most researchers have been mining valuable genes and alleles from teosinte or *Tripsacum* using the abovementioned genetic bridges. Attempts to develop a novel bridge parent that permits the movement of exotic genes from wild relatives into maize have been sluggish in the 21^st^ century. Are there other ways to exploit these treasure troves for maize improvement? As Harlan and De Wet (1977) noted, “It is now apparent that the choice of the *Tripsacum* parent is critical and that events occurring in the early backcrossing generations largely determine the ultimate outcome.” Recently, Iqbal *et al.* (2019), from our laboratory, reported a promising bridge (MTP) in which the tetraploid *Tripsacum* and tetraploid perennial teosinte coexist under a maize background and confirmed the value of this bridge for improving maize diversity. This work is a successful attempt to open up a new pathway to broaden the maize germplasm. Moreover, MTP was also used as a seed parent for breeding perennial forage maize (Li *et al.* 2015).

Given the previous naming rule, tripsacoid was defined as the characters introgressed from *Tripsacum* into maize (Anderson and Erickson 1941; Stalker *et al.* 1977; Harlan and De Wet 1977). The newly synthesized allohexaploid (MTP) encompasses the genomes from the genera *Tripsacum* and *Zea*; thus, MTP will be subsequently referred to as tripsazea. Here, we depict its synthesis, morphological characteristics, and chromosomal and reproductive behavior to better exploit tripsazea.

## Materials and Methods

### Material synthesis

The parental materials used in this study were a maize-*Tripsacum* hybrid (2*n* = 4*x* = 20Zm + 36Td, genome = MMTT, accession No. H278) and a tetraploid *Z*. *perennis* (2*n* = 4*x* = 40Zp, PPPP, 9475) from CIMMYT. The maize-*Tripsacum*, obtained from the United States Department of Agriculture (USDA), was kindly provided by Dr. Maolin Zhao in 2005, Beijing Academy of Agricultural and Forestry Sciences, Beijing, China. Employment of the material was found in previous studies (Kindiger *et al.* 1996a and 1996b). Original diploid *Z*. *mays* and tetraploid *T*. *dactyloides* of maize-*Tripsacum* were not available. Fortunately, tetraploid *T*. *dactyloides* (2*n* = 4*x* = 72Td, TTTT, TZ07) and tetraploid maize (2*n* = 4*x* = 40Zm, MMMM, V182) were introduced by the USDA for species comparison. In 2005, the trispecific hybrids were generated by crossing the maize-*Tripsacum* hybrid with 9475 by shortening silks and repeated pollination by Prof. Qilin Tang. Subsequently, these trihybrids with different chromosomal numbers were successfully synthetized (see below). Chromosome counting of these trihybrids was performed using the method of Cheng *et al.* (2016). Of all the trihybrids, a unique hybrid (*i.e.*, tripsazea) has perenniality, which offered us the opportunity to execute the following experiment.

### Morphological characteristics

A field trial was established at Chongzhou Agricultural Research Station of Sichuan Agricultural University (30°33′N, 103°38′E, elevation 523 m), Sichuan, southwestern China. The average annual precipitation is 1012.4 mm, with an extremely high temperature of 37.7° in the summer and an extremely low temperature of -3° in the winter. On April 20, 2017, plantlets (tripsazea, TZ07 and 9475) for the planting were excavated from the mother stands established in 2016 at Chongzhou. The plantlets used consisted of a single shoot with three leaves and lateral roots. A wide distance of 1.0 m between plants and 1.5 m between rows was adopted. On the same day, V182 seedlings with three leaves were also transplanted at the same density for comparison. Qualitative traits were recorded, and metric traits were measured 10 times for each material.

### Genome change

The nuclear DNA content was determined from fresh leaves by flow cytometry following Kaushal *et al.* (2010). The nuclei isolated from *Z*. *mays* (inbred line, B73) were used as an internal standard (4.85 pg 2C^−1^) (Gui *et al.* 2007) for the determination of the relative 2C DNA content of tripsazea and its three parents. Each material was analyzed three times with a CytoFLEX flow cytometer (Beckman Coulter).

### Chromosome behavior

The root tips from tripsazea were pretreated in α-bromonaphthalene at room temperature for 3 h, fixed in 3:1 (v/v) ethanol/glacial acetic acid (Carnoy’s fixative) at -4° overnight, and transferred to 70% ethanol at -4° for storage. After placing the root tips in a mixture of 6% cellulase (R-10, Yakult) and 1% pectinase (Y-23, Yakult) for 3 h at 37°, the roots were washed in distilled water. We transferred the root tips to a clean slide and then dropped a drop of Carnoy’s fixative with a fine needle and immediately dried the slide over an alcohol flame. The dried spreads were observed by phase-contrast light microscopy to screen spreads with well-spread metaphase cells. The spreads screened were saved at -20° for mitotic profiling. The PMCs from tripsazea were collected, treated in Carnoy’s fixative overnight, and then transferred to 70% ethanol at -4° for meiotic profiling. The PMC chromosome preparation followed the method mentioned above.

Genomic *in situ* hybridization (GISH) was used to distinguish the M (*Z*. *mays*), T (*T*. *dactyloides*) and P (*Z*. *perennis*) genomes in tripsazea. Total DNA was extracted from young leaves of tetraploid maize (V182) and *Z*. *perennis* (9475) through a modified 2× CTAB method (Fu *et al.* 2015). According to the manufacturer’s protocols, the V182 DNA and the 9475 DNA were labeled with DIG-Nick Translation Mix (Roche) and BIOTIN-Nick Translation Mix (Roche), respectively. After placing the slides selected in 50 ml RNase A solution (Solarbio, 0.1 μg/ml in 2× SSC) for 60 min at 37°, the slides were washed twice in 2× SSC for 5 min each at room temperature. Slides were denatured in 70% deionized formamide (FAD) at 70° for 2 min and were quenched in ice-cold (-20°) 70% ethanol followed by dehydration (90% and 100% ethanol for 5 min each at room temperature). The hybridization mixture contained 50% FAD, 10% dextransulfate, 2× SSC, 0.5% SDS, 10 μg salmon sperm DNA, and 18 ng/μl for each probe DNA. The mixture was incubated at 80° for 10 min and then cooled with ice for 10 min. Subsequently, 45 μl of the mixture was added to each slide and incubated at 37° in a container overnight. After hybridization, slides were washed in 20% FAD for 15 min, and stringent washing followed by 2× SSC and 0.1× SSC at 42° for 15 min each was performed. The slides were then washed in 0.1% TritonX-100 for 5 min and 1× PBS three times (5 min each) at room temperature. After drying at room temperature, each slide was incubated with 47.7 μl anti-digoxigenin-fluorescein mix (Roche) and CY-3 fluorescein (Sigma) for 1 h at 37° in the dark and was then washed with 1× PBS three times. Finally, the slides were counterstained with DAPI and observed with an Olympus fluorescence microscope (BX-61). Images were taken with a Media Cybernetics CCD 700 (Charge Coupled Device) and Image Pro Plus 6.0 (Media Cybernetics, Inc.).

### Reproductive behavior

Flowering dates of each material were observed under field conditions in Chongzhou. The percentage of stainable pollen grains was calculated to measure pollen fertility. To investigate the fertility of the female gametes, we also tried to stimulate embryo development in tripsazea by dusting its silk with pollen from cultivated maize (Mo17). The seed setting rate was expressed by the number of seeds set/florets pollinated with the pollen of Mo17. Several teosintes, *i.e.*, *Z*. *mexicana*, *Z*. *perennis*, *Z*. *nicaraguensis* and *Z*. *luxurians*, were also used as pollen donors to determine whether tripsazea can produce seeds.

### Data availability

All data generated during the study appear in the article.

## Results

### Crossing maize-Tripsacum and *Z*. perennis

**Figure 1:**
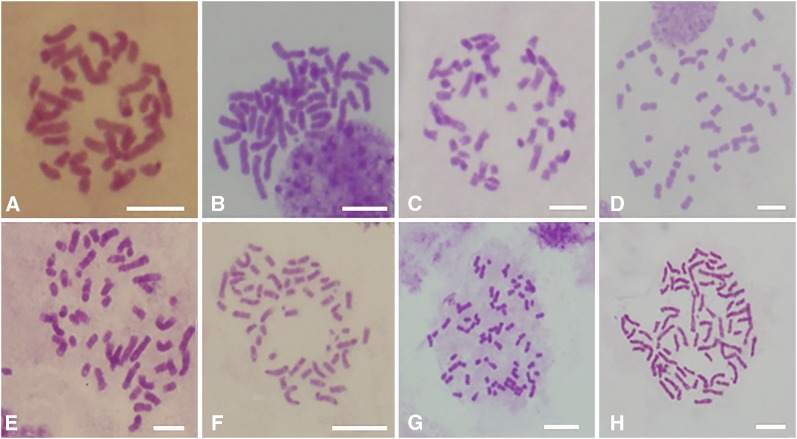
Seven types of chromosome numbers in trihybrids. The chromosome number in trihybrids included (A) 2*n* = 36, (B) 2*n* = 46, (C) 2*n* = 50, (D) 2*n* = 52, (E) 2*n* = 52, (F) 2*n* = 54, (G) 2*n* = 72, and (H) tripsazea, 2*n* = 74. Scale bars, 10 μm.

We pollinated 121 female spikelets of H278 with pollen from 9475 and obtained 50 seeds forming 37 seedlings. There were 10 seeds with double embryos. Trihybrids presented diverse morphological characters. Trihybrids were significantly higher than tetraploid maize in terms of tiller number and lateral branch number and were wider than *T*. *dactyloides* and *Z*. *perennis* in terms of stem diameter and leaf width, and most traits were intermediate among three parents. The expected chromosome number of artificial trihybrids is 48 chromosomes, including 10Zm, 18Td and 20Zp. However, 18 trihybrids with chromosome numbers ranging from 36 to 74 were observed, and seven types of chromosome numbers were observed in the trihybrids (Figure 1). Their chromosome numbers were 36 (1 plant), 46 (2 plants), 50 (2 plants), 52 (8 plants), 54 (3 plants), 72 (1 plant) and 74 (1 plant). The percentage of stainable pollen of trihybrids ranged from 0 to 9.69%, which was significantly lower than that of the three parents. Because H278 was dead in nature, we could not duplicate the synthesis process. In addition to perennial material, we lost other trihybrids that are annual or weakly perennial. No more information was obtained for lost trihybrids. Therefore, we must turn our attention to the sole survivor. The survivor (tripsazea) has 74 chromosomes (Figure 1H). Theoretically, it is an allohexaploid with two chromosomes missing. Iqbal *et al.* (2019) affirmed that the chromosome composition of tripsazea is 2*n* = 6*x* = 20Zm + 34Td + 20Zp with two *Tripsacum* chromosomes missing based on McGISH with a Cent-C probe.

### Morphological characteristics of tripsazea

**Figure 2:**
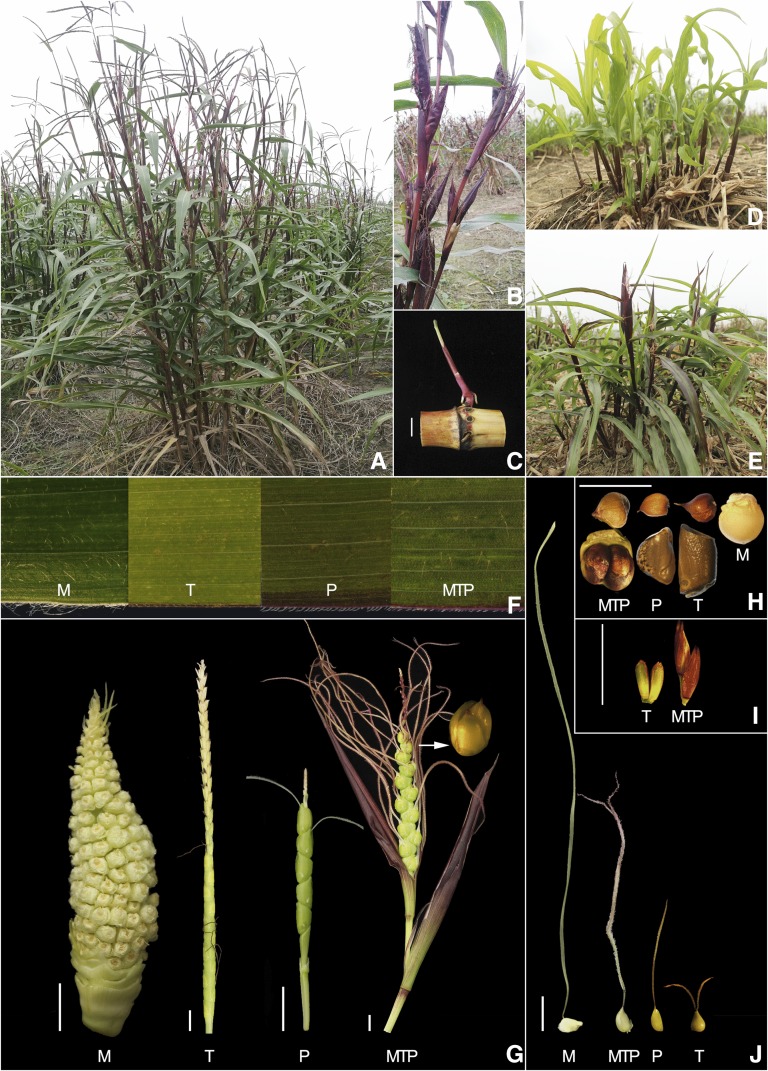
Morphology of tripsazea and comparison of its several morphological characters with three parental species. (A) One-year-old tripsazea plant by stem node propagation. (B) Tripsazea ears from axillary bud differentiation, branching of lateral inflorescence. (C) A tripsazea seedling from the axillary bud. (D) Regenerated tripsazea plants after mowing. (E) Flowering of tripsazea plants induced by short-day exposure. (F) Mature leaves from the hybrid and its parents (area 1 cm^2^). The ear (G), seed (H), male spikelet (I) and silk (J) from the hybrid (tripsazea: MTP) and its parental species (M, T, and P designations refer to *Z*. *mays*, *T*. *dactyloides*, and *Z*. *perennis*, respectively). Scale bars, 1 cm.

The morphological characteristics of tripsazea with its three parental species are shown in Figure 2, and a comparison of some morphological traits is also shown in Table 1. Trispecific tripsazea is a tall and erect bunchgrass with a high tiller number that grows more prolifically than its three parents. At first glance, tripsazea looks more like *Z*. *perennis* than maize and *Tripsacum*. However, it is taller, with a longer leaf length, greater leaf width, wider stem diameter, and a lower tiller number than *Z*. *perennis*. Tripsazea exhibits the perennial characteristics of both parents, *viz*., *T*. *dactyloides* and *Z. perennis*. Tripsazea, such as *Z*. *perennis*, can be propagated through the stand and aboveground stem node. The silks are longer than those of *Z. perennis* and *T*. *dactyloides* and shorter than those of *Z*. *mays*. Similar to *T*. *dactyloides*, some styles of tripsazea have a bifurcated tip. Among the 4 materials tested, the kernels of tetraploid maize (V182) are naked and the largest in size. The kernels of *T*. *dactyloides* and *Z. perennis*, smaller than V182 in size, are enwrapped in a hard fruitcase consisting of the rachis and the outer glume. However, the kernels of tripsazea showed an intermediate type compared to the parents, with moderate size, and are partially enclosed by glumes. Tripsazea plants had senesced by December. Tripsazea seedlings that regenerate in autumn can undergo low temperature (>5°) and die after frost killing. Normally, five to ten ears are produced on a culm during a flowering period, and plants produce 150-200 ears twice annually. Every culm produces 5-7 branches, and the top and node branches grow the female spike; each ear has 8-18 ovules with opposite rows of paired seeds. Tripsazea possesses features of perennation and agamic propagation, making it a good genetic material for continuous research.

**Table 1:** Morphological characteristics of the tripsazea along with its parental species

CHARACTER	*Z*. *mays*	*T*. *dactyloides*	*Z*. *perennis*	TRIPSAZEA
Life cycle	Annual	Perennial	Perennial	Perennial
Rhizome	Absent	Present	Present	Absent
Female spike enclosed in husks	Enclosed	Naked	Enclosed	Enclosed
Pedicelle on second male spikelet	Pedicelled	Sessile	Pedicelled	Pedicelled
Seeds enclosed in shell	Naked	Enclosed	Enclosed	Half-naked
Tiller number	0.30 ± 0.67d	49.10 ± 12.08a	38.90 ± 9.80b	12.60 ± 4.40c
Leaf width (cm)	5.87 ± 0.44a	1.81 ± 0.17d	2.67 ± 0.45c	4.50 ± 0.64b
Leaf Length (cm)	58.20 ± 7.36b	112.07 ± 25.98a	36.55 ± 8.20c	59.57 ± 10.87b
No. ears per stem	1.00 ± 0.00c	8.70 ± 2.36b	7.10 ± 1.66b	14.00 ± 5.19a
No. ovules per ear	141.40 ± 24.14a	10.00 ± 1.76b	6.20 ± 0.42b	15.30 ± 4.22b
No. branches per tassel	8.0 ± 1.83a	Absent	2.5 ± 1.08c	4.4 ± 1.43b
Branching of lateral inflorescence	Absent	3.30 ± 1.16b	4.90 ± 2.42a	6.20 ± 0.79a
Silk length (mm)	15.93 ± 2.35a	2.24 ± 0.32d	4.91 ± 1.08c	9.70 ± 1.95b
Height of main tiller (cm)	112.00 ± 8.63d	211.60 ± 33.26b	183.60 ± 12.08c	250.20 ± 22.20a
Stem diameter (mm)	16.24 ± 2.54a	10.45 ± 1.53b	12.07 ± 2.65b	16.77 ± 1.94a
No. row functional pistillate spikelets	8, 10, or 12	2	2	4

Data are mean ± SD. Different letters in a row mean significant difference between means at α = 0.05 level according to Duncan’s multiple range test.

### Genome changes in tripsazea

**Figure 3:**
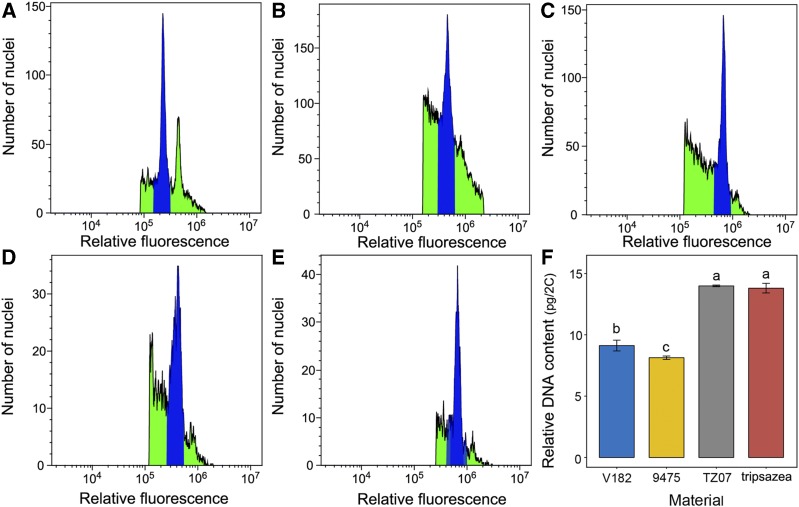
FCM histograms from leaves of tripsazea and its three parental species, using maize inbred line (B73) as an internal standard: (A) B73, (B) V182, (C) TZ07, (D) 9475, (E) tripsazea, and (F) comparison of the relative DNA content (data shown as mean and S.D., n = 3; different lowercase letters indicate statistically significant difference at the α = 0.05 level according to Duncan’s multiple range test).

After the formation of polyploid, the genome size often shows two results: no change, or genomic fragments (or some genes) are lost. As expected, tripsazea showed the latter situation owing to the loss of two chromosomes from *Tripsacum* (Figure 3). The DNA content of the parent was 9.12 ± 0.44 pg 2C^-1^ in tetraploid *Z*. *mays*, 13.99 ± 0.07 pg 2C^-1^ in *T*. *dactyloides*, and 8.13 ± 0.14 pg 2C^-1^ in *Z*. *perennis*. The results showed that the DNA content could be utilized as a marker to distinguish the three parents. However, the genome size of tripsazea was 13.81 ± 0.39 pg 2C^-1^, which was significantly lower than the expected value (15.62 pg 2C^-1^), indicating genome downsizing during allohexaploidization among the three species.

### Chromosome behavior of tripsazea

**Figure 4:**
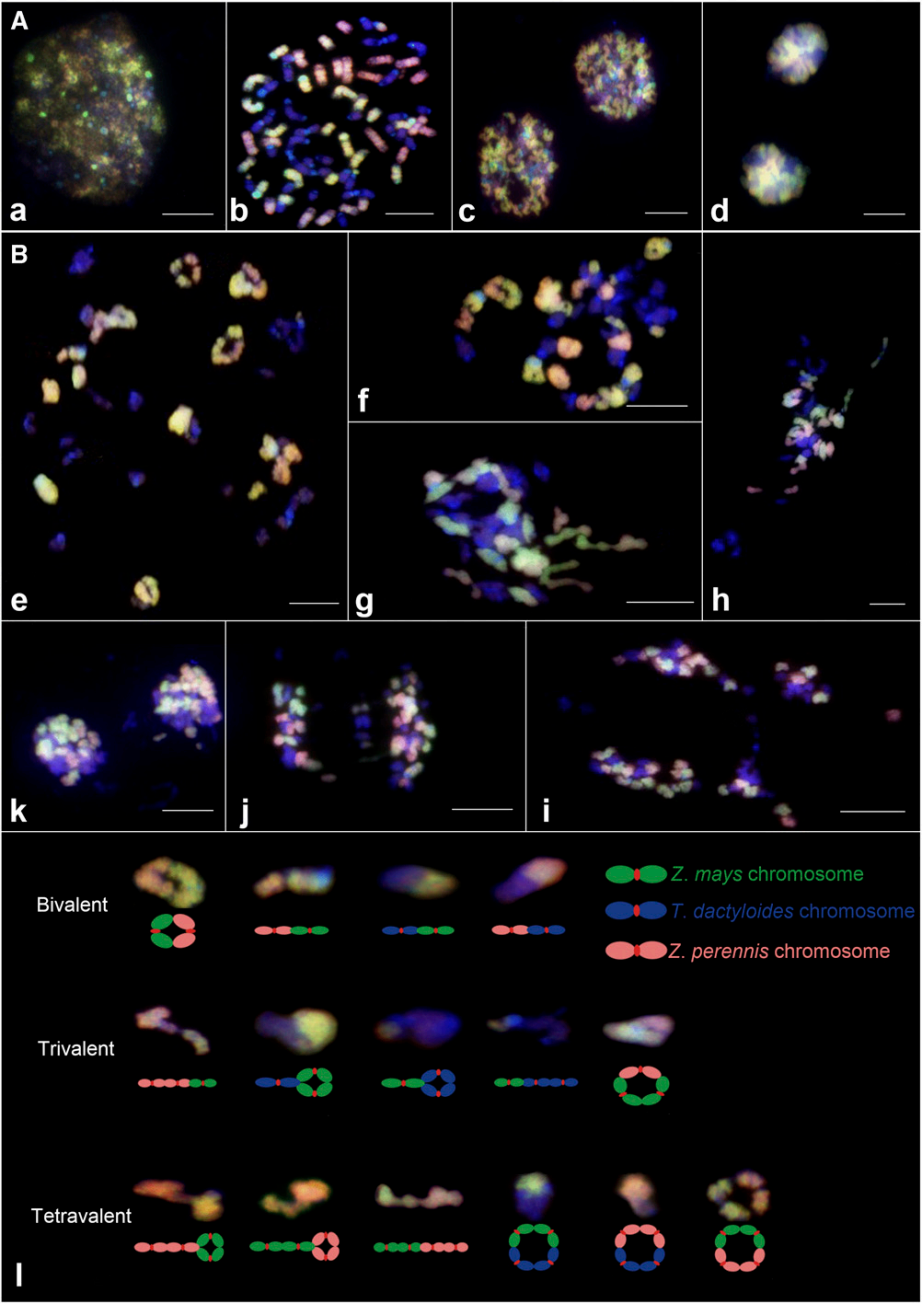
The mitotic (panel A) and meiotic behavior (panel B) of tripsazea. The prophase (a), metaphase (b), anaphase (c) and telophase (d) of mitosis. Diakinesis I (e, f, and g), metaphase I (h), anaphase I (i and j) and telophase (k) of meiosis and observed allosyndetic associations and their diagram (l). Yellow (or green), pink and blue signals were from the maize, *Z*. *perennis* and *T*. *dactyloides* genomes, respectively. Scale bars, 10 µm.

The mitotic observation of tripsazea showed that its chromosomes from three genomes did not exhibit irregular mitotic behavior (Figure 4A). There were no lagging chromosomes or chromosome bridges, indicating that the mitotic behavior of tripsazea was stable and was not affected by the addition of the external genome.

Meiotic analysis of tripsazea revealed the occurrence of 4.24I + 27.19II + 0.79III + 3.25IV at diakinesis I in average chromosome association per pollen mother cell (Table 2 and Figure 4B). On average, 73.49%, 17.57%, 5.73%, and 3.20% of the chromosomes in PMCs paired as bivalents, tetravalents, univalents and trivalents, respectively. Multivalence with a higher number was not observed. In our observations, we detected that 5.73%, 75.84% and 18.42% of the chromosomes participated in no, homologous and heterologous pairing, respectively. For heterozygosis, allosynapsis between the M- and P-genomes was dominant (16.43%). All multivalent (≤4) configurations involving the M-genome and P-genome were present, and the most frequent configuration was 1.53 II_mp_ + 1.84 IV_mmpp_. Allosyndetic pairing between the M- and T-genomes was minor (1.59%), and the T- and P-genome pairing was the lowest (0.39%). Some multivalent configurations involving the T-genome were not observed, including III_tpp_, IV_mmmt_, IV_tppp_, IV_mttt_, and IV_tttp_. These results suggested that the homology between *Z*. *mays* and *Z*. *perennis* was the highest, followed by *Z*. *mays* and *T*. *dactyloides*, and the homology between *T*. *dactyloides* and *Z*. *perennis* was the lowest. All of these allosynapses revealed that these chromosomes have partial homology. Although a few intergenomic multivalents (Figure 4l) were formed, introgression of specific target genes from *T*. *dactyloides* or *Z*. *perennis* into *Z*. *mays* and vice versa may be foreseeable.

**Table 2:** Cytological features of PMCs (n = 126) in tripsazea at diakinesis I

CHROMOSOME ASSOCIATION		NUMBER (PERCENTAGE) OF PMCS WITH CORRESPONDING VALENT	MEIOTIC CONFIGURATION		
			AVERAGE	MIN.	MAX.
Univalent	m	83 (65.87)	1.04	0	6
	t	89 (70.63)	2.24	2	12
	p	63 (50.00)	0.96	0	6
	**I**		**4.24**		
Bivalent	mm	126 (100.0)	5.29	2	10
	tt	126 (100.0)	14.55	9	17
	pp	126 (100.0)	5.59	1	9
	mt	24 (19.05)	0.19	0	1
	mp	81 (64.29)	1.53	2	6
	tp	5 (3.97)	0.04	0	1
	**II**		**27.19**		
Trivalent	mmm	12 (9.52)	0.09	0	1
	ttt	9 (7.14)	0.07	0	1
	ppp	3 (2.38)	0.02	0	1
	mmt	17 (13.49)	0.14	0	1
	mtt	3 (2.38)	0.02	0	1
	mmp	17 (13.49)	0.20	0	3
	mpp	24 (19.05)	0.22	0	2
	ttp	4 (3.17)	0.03	0	1
	tpp	—	—	—	—
	**III**		**0.79**		
Tetravalent	mmmm	32 (25.40)	0.27	0	2
	tttt	20 (15.87)	0.53	0	4
	pppp	40 (31.75)	0.38	0	2
	mmmt	—	—	—	—
	mmtt	11 (8.73)	0.08	0	1
	mttt	—	—	—	—
	mmmp	8 (6.35)	0.06	0	1
	mmpp	117 (92.86)	1.84	0	5
	mppp	8 (6.35)	0.06	0	1
	tttp	—	—	—	—
	ttpp	4 (3.17)	0.03	0	1
	tppp	—	—	—	—
	**IV**		**3.25**		

Chromosome complements from respective species: m–*Z*. *mays*, t–*T*. *dactyloides*, p–*Z*. *perennis*; each alphabet denotes a univalent dose.

Chromosomal segregation of tripsazea in anaphase I was also observed (Figure 4i, j and k). The maximum chromosome number of *Tripsacum* was 18 in daughter cells, the minimum chromosome number was 12, and the average chromosome number was 15.7. The maximum daughter cell chromosome number of maize (*Z*. *perennis*) was 13 (13), and the minimum was 6 (6), with an average of 9.6 (9.5). The maximum number of *Tripsacum* lagging chromosomes was 8, the minimum was one with an average of 4.1, and all observed cells had *Tripsacum* lagging chromosomes. In *Z*. *mays* and *Z*. *perennis*, 50% of the cells tested showed lagging chromosomes. The average (range) of lagging chromosomes was 1.4 (1-3) in *Z*. *mays* and 1.4 (1-2) in *Z*. *perennis*. The results suggested that *Tripsacum* chromosomes might be more easily lost in gametogenesis compared with *Z*. *mays* and *Z*. *perennis*.

### Reproductive behavior of tripsazea

Tripsazea was highly photoperiod sensitive and biannually flowered under short-day conditions (first in April-May and again in September-December). The immediate parent 9475 flowered annually during the months of October-December. The flowering period of *T*. *dactyloides* began in May and extended into October. Trihybrid tripsazea showed extremely low male fertility (0.16% stainable pollen). Despite the low temperature during the flowering period of tripsazea, it can produce mature seeds after open pollination conditions, indicating that the female ear has partial fertility. When Mo17 was used as a pollen parent, the seed setting rate of tripsazea was 3.16% under artificial pollination conditions. Seeds could be obtained with pollen from *Z*. *perennis*, *Z*. *nicaraguensis*, *Z*. *mexicana* and *Z*. *luxurians*, but the seed setting rate was not investigated.

## Discussion

### Previous trihybrids involving maize, Tripsacum and teosinte

Initially, a triple hybrid [(*Z*. *mays* × *T*. *dactyloides*) × *Z*. *mexicana*, 2*n* = 3*x* = 10Zm + 18Td + 10Ze] created by Mangelsdorf and Reeves (1935) was sterile and annual without stainable pollen and with an extremely low seed-setting rate (maize pollen 0.96% and *Z*. *mexicana* pollen 0%). Newell and De Wet (1973) obtained a series of triploid plants from the cross of *Z*. *mays* × *T*. *dactyloides* hybrids and *Z*. *mexicana* or *Z*. *perennis*. Walton Galinat developed a perennial trihybrid (2*n* = 3*x* = 10Zm + 18Td + 10Zd) by crossing a colchicine-doubled allodiploid [*Z*. *mays* (MM) × *T*. *dactyloides* (TT)] with *Z*. *diploperennis* (Lamb and Birchler 2006). A perennial genotype, *i.e.*, tripsazea, was generated in our laboratory by pollinating a maize-*Tripsacum* hybrid (MMTT) with *Z*. *perennis*.

Morphologically, the phenotypic change of these trihybrids is similar to maize, including enhanced photoperiod sensitivity, increased number of tillers and branches caused by the gene *teosinte branched1* (*tb1*), and transmutative ear architecture dominated by the gene *teosinte glume architecture* (*tga1*) (Hufford *et al.* 2012). Several hybridization pathways, *i.e.*, n + n, 2n + n, 2n, and an irregular type, were reported in the formation of hybrids containing *Tripsacum* and maize (Newell and De Wet 1973). In the reported dihybrids of maize and *Tripsacum*, the range of chromosome numbers was 21-92 (maize chromosomes 6-50 and *Tripsacum* chromosomes 1-72) owing to the facultative apomixis of *T*. *dactyloides* in the maize-*Tripsacum* background (Harlan and De Wet 1977). Therefore, the synthesis of trihybrids with different chromosome numbers (36-74) in our cross was not surprising.

According to the genomic composition of tripsazea, we present three speculations for its formation. First, the maize-*Tripsacum* hybrid may have had 54 chromosomes in somatic cells. In this situation, tripsazea was a product of a 2n + n mating whereby an unreduced egg of tetraploid maize-*Tripsacum* was fertilized by a sperm nucleus of *Z*. *perennis*. Second, the maize-*Tripsacum* hybrid with 56 chromosomes produced a gamete with complete genomes of maize and incomplete genomes of *Tripsacum*. Third, tripsazea was generated by fertilization between a 2n female gamete from maize-*Tripsacum* with 56 chromosomes and an n male gamete from *Z*. *perennis* with subsequent 2 Td chromosomes elimination in early embryogenesis.

### Chromosome pairing within genomes in hybrids involving maize, Tripsacum or *Z*. perennis

Numerous cytological findings have shown that maize (2*n* = 20) forms almost 10 bivalents at meiosis (Poggio *et al.* 2000; Iqbal *et al.* 2018). When diploid and tetraploid maize was pollinated with *Z*. *perennis*, in the P-genome from dihybrids, the proportion of chromosomes participating in heterologous pairing was 49.75% for an allotriploid (10Zm + 20Zp) and 47.70% for an allotetraploid (20Zm + 20Zp) (Iqbal *et al.* 2018). However, intergenomic pairing in maize-*Tripsacum* dihybrids is always rare (De Wet and Harlan 1974) and dependent on the genetic background (Harlan *et al.* 1970). For example, the basic genomes of maize (*x* = 10) and *T*. *dactyloides* (*x* = 18) have little genomic affinity for each other in hybrids (10Zm + 18Td), and one (or at most two) Zm chromosome can occasionally form loose bivalents with Td chromosomes during meiosis (De Wet and Harlan 1974). In most hybrids (10Zm and 36Td), Td chromosomes associate into 18 bivalents; in a few hybrids, 1-4 Zm chromosomes occasionally associate with Td chromosomes to form bivalents and trivalents (De Wet and Harlan 1974). These hybrids (20Zm and 36Td) usually present 18 *Tripsacum* bivalents; hybrids with 10Zm + 72Td show 36 *Tripsacum* bivalents, and 8 Zm chromosomes have pairing affinities with Td chromosomes in rare cases (De Wet and Harlan 1974).

When the chromosomes of *Z*. *perennis* were inserted into the maize-*Tripsacum* dihybrid, trihybrid tripsazea formed more allosyndetic associations than maize-*Tripsacum* and less allosyndetic associations than maize-*Z*. *perennis* during meiosis. The percentage of allosyndetic-association chromosomes was 18.42% for tripsazea, 51.17% for an allotriploid (10Zm + 20Zp) (Iqbal *et al.* 2018), 47.70% for an allotetraploid (20Zm + 20Zp) (Iqbal *et al.* 2018), and almost zero for maize-*Tripsacum* (20Zm + 36Td) (De Wet and Harlan 1974). Among allosyndetic pairings in tripsazea, pairings between the Zm and Zp chromosomes were the highest (16.43%), followed by Zm and Td chromosomes (1.59%), with the lowest pairings (0.39%) for Zp and Td chromosomes. When the valents with Td (Zp) were excluded from tripsazea, the percentage of allosyndetic-association chromosomes was 30.40% (2.19%). In the P-genome (T-genome) from tripsazea, the percentage of chromosome participating in heterologous pairing was 31.11% (1.70%). These results, together with previous research (De Wet and Harlan 1974; Molina *et al.* 2006; Iqbal *et al.* 2018), revealed that a) *T*. *dactyloides* is more closely related to *Z*. *mays* than *Z*. *perennis*, b) three genomes sharing one nucleus led to a reduction in heterologous pairings in comparison with an intrageneric dihybrid, and c) intergenomic pairing seemed to be higher but still rare in the trihybrid in comparison with its intergeneric dihybrid parent.

### Tapping the maize secondary genepool via tripsazea

To our knowledge, previously synthetic trihybrids aimed at understanding the evolutionary relationship among the three species and were seldom applied to maize improvement. Prior breeding efforts for transferring valuable genes lost during domestication into cultivated maize were focused on digenomic hybrids derived from maize × teosinte hybrids and maize × *Tripsacum* hybrids. Although *T*. *dactyloides* has a more diverse genetic variation than teosinte, its gene pool has not been sufficiently tapped. First, an efficient *Tripsacum* bridge is a prerequisite for mining *Tripsacum* genetic resources. However, it is not easy to synthesize *Tripsacum* bridges by traditional crossing, even when combined with the embryo rescue technique. In a recent study, a total of 94,643 seeds from 54 eastern gamagrass populations exposed in proximity to glyphosate-tolerant maize fields were evaluated by a glyphosate-tolerant test and transgene marker (Lee *et al.* 2017). Their results indicated that natural hybridization between *Tripsacum* and maize is impossible. Of the formerly synthesized bridges, many are ephemeral, making them difficult to conserve and produce a large number of seeds of first backcrossed progeny. Meanwhile, the process of introducing genetic diversity from *Tripsacum* into maize and the exclusion of linkage drag genes require a large amount of time, manpower and material resources. Despite these difficulties, given the GM debate and ineffectiveness of GM technology for some traits (such as drought tolerance, see Gilbert 2014), this approach may be our best current strategy for introducing some alien genes into maize.

According to a review by Harlan and De Wet (1977), allodiploid (MT) is regarded as a hopeless crossing bridge for introgressing tripsacoid characters from *Tripsacum* into maize; allotriploid (MTT) usually results in recovered maize without or with little *Tripsacum* chromatin. Does a trihybrid incorporating the genetic resources of the three species have the potential to advance maize breeding? First, it may introduce the excellent traits of *Z*. *perennis* and *T*. *dactyloides* into maize. Second, it may promote or retard the introgression of *Tripsacum* genes into maize or favor the introgression of *Z*. *perennis* into maize because much higher homologous pairing was found between maize and tetraploid teosinte than between maize and *Tripsacum*. A high heterozygosity of tripsazea may provide a valuable bridge for chromosomal translocation to occur. Third, utilization of an allopolyploid as a bridge in maize backcrosses to produce epigenetic variation may also offer an opportunity for genetic improvement of maize.

By backcrossing tripsazea with Mo17 (Iqbal *et al*. 2019), progenies with different chromosome numbers emerged with three types of chromosomal cross-talk, including Zm-Zp (91.51% of all translocations), Zm-Td (4.72%), and Zp-Td (3.77%). Among the analyzed descendants, an additional maize-*Tripsacum* (2*n* = 20Zm + 1Td) line was obtained. In mating behavior, diversity of reproductive modes in tripsazea was found, including 2n, 2n + n, n + n and an irregular type. With respect to morphological characteristics, plant phenotypes of the backcrossed generation were diverse in terms of tiller number, lateral branch number, stem diameter, plant height, and ear traits. Therefore, tripsazea could serve as a conduit for gene flow from *T*. *dactyloides* and/or *Z*. *perennis* into maize.

### Breeding perennial forage crops by tripsazea

Perennial forage maize has good forage quality, palatability and perenniality, making it a useful forage crop. Perennial teosintes, *viz*., *Z*. *perennis* and *Z*. *diploperennis*, are the basic versions of perennial forage maize. Initial success in breeding perennial forage maize was a single-cross hybrid of the *Z*. *mays-Z*. *perennis* substitution line (2*n* = 20) and *Z*. *perennis*, combining the tolerance to abiotic stresses and perenniality obtained from the *Z*. *perennis* parent with the rapid growth from the maize parent (Ren *et al.* 2007). Here, we have reason to expect another success via tripsazea. We integrated *Z*. *perennis* into maize-*Tripsacum* to improve the biomass of tripsazea. Tripsazea is an immortal genotype that combines perenniality and winterhardiness obtained from *T*. *dactyloides* and *Z*. *perennis*. Due to its high pollen sterility and low seed setting rate, it is impossible to develop perennial forage maize through seed. However, tripsazea and perennial teosintes (*Z*. *diploperennis* and *Z*. *perennis*), possessing vegetative propagation ability, allow us to breed agronomically superior clonal varieties with higher biomass and stress resistance by using tripsazea as a female parent in crosses with perennial teosintes.

**Figure 5:**
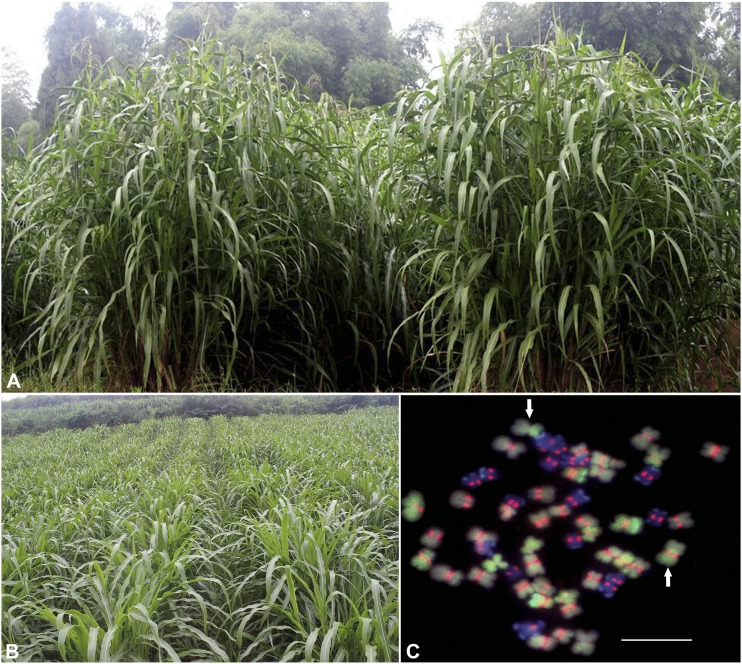
Field-grown perennial forage maize cv. Yu5 (A and B) and its chromosomal constitution (C) (Li *et al.* 2015). Blue, gray blue, green, and red colors represent *T*. *dactyloides*, *Z*. *perennis*, *Z*. *mays* chromosomes and Cent-C probes, respectively. Arrows indicate translocation chromosomes between *Z*. *mays* and *Z*. *perennis*. Scale bar, 10 µm.

As expected, a vigorous perennial clone (Figure 5A), ‘Yuqilin 58’ (now known as ‘Yucao No. 5′, Yu5), was successfully obtained (Li *et al.* 2015). These authors reported that this clone was synthetized by crossing tripsazea with *Z*. *perennis* by an n + n mating and that Yu5 possesses 58 chromosomes (Figure 5B), with an aneuploidy representing 11, 17 and 28 chromosomes from *Z*. *mays*, *T*. *dactyloides*, *Z*. *perennis*, and 2 Zm-Zp translocation chromosomes, respectively. The vegetative propagation of Yu5 by ramets and stems has been shown by Li *et al.* (2015): over 2 years of their trial in Chengdu, China, Yu5 at a 1.0 m × 1.0 m spacing achieved 22.08 and 14.39 Mg ha^-1^ of dry matter yield in the first year and second year, respectively. In 2019, Yu5 was approved as a forage variety by the National Grass Variety Approval Committee.

### Tripsazea as a model for allopolyploid study

In addition to the promising breeding application mentioned above, tripsazea has specific academic significance in polyploid research. Tripsazea and its perennial parents are asexual clones, and the tetraploid maize parent is conserved by seed, allowing us to continuously excavate information on the incipient stage after the formation of the allopolyploid. We confirmed the chromosome and DNA sequence elimination of polyploidization. Undoubtedly, tripsazea will provide an intergeneric material to reveal cross-genus regulation for gene and epigenetic expression patterns of allopolyploids to investigate the postpolyploid genomic evolution and to examine duplicate gene fate, among other applications. These studies will bring new insights into allopolyploid evolution and its relationship with maize domestication and improvement.

## Conclusions

In this study, we used a synthetic allotetraploid (from the cross between tetraploid *Z*. *mays* and tetraploid *T*. *dactyloides*) as the seed parent in a cross with *Z*. *perennis* to generate trihybrids with different chromosome numbers. Of these hybrids, a superior genetic resource (tripsazea), which has two sets of genomes from each parent species, was produced. Many genetic bridges used in previous studies are ephemeral and were used once. Tripsazea is perennial and is maintained by propagules, making it easy for reuse. Tripsazea can be readily crossed with maize and produce partially fertile hybrids. Through the trigenomic bridge as a female parent, maize breeders may access almost all the genetic resources in the wild relatives for genetic improvement of maize and the development of new perennial forage crops. In conclusion, we offer a potential material and pathway to circumvent the *Tripsacum*-maize crossing barrier and to introduce alien genes from *T*. *dactyloides* and/or *Z*. *perennis* into maize.
